# Vectorial characterization of surface wave via one-dimensional photonic-atomic structure

**DOI:** 10.1038/s41598-023-49324-6

**Published:** 2023-12-08

**Authors:** M. Asadolah Salmanpour, M. Mosleh, S. M. Hamidi

**Affiliations:** Magneto-Plasmonic Lab, Laser and Plasma Research Institute, Shahid Beheshti University, Tehran, Iran

**Keywords:** Materials science, Condensed-matter physics, Nanoscale materials

## Abstract

Quantitative assessment of polarization properties of waves opens up the way for effective exploitation of them in many amazing applications. Tamm surface waves (TSW) that propagate on the interface of periodic dielectric media are proposed for many applications in numerous reports. The polarization state of TSW is not simply intuitive and would not be extracted from reflection spectra. Here considering orientation sensitive nature of the interaction between polarized electromagnetic wave and atom, we try to quantitatively characterize the polarization state of TSWs, excited on the surface of the 1D photonic crystal. To do this we performed direct contact between TSW and rubidium atomic gas by fabrication of a one-dimensional photonic crystal-atomic vapor cell and applied a moderate external magnetic field to create geometrical meaning and a sense of directionality to dark lines in reflection intensity. Our experimental results indicate that transition lines in the reflection spectrum of our hybrid system modify dependent on the orientation of the applied magnetic field and the transverse spin of TSW. We have used these changes to redefine the geometry of Voigt and Faraday for evanescent waves, especially Tamm surface waves**.** In the end, we performed simple mathematical operations on absorption spectra and extract the ratio of longitudinal and transverse electric field components of the polarization vector of TSW equal to $\frac{2}{5}$.

## Introduction

In the late seventies, the existence of surface electromagnetic waves on the interface between the 1D photonic crystal and an external medium was experimentally investigated^1–3^. After that, extensive studies have been conducted on fundamental principles governing the emergence and behavior of ESWs at the surface of 1D and 2D-photonic crystals^4–7^. In the last two decades, the interest in investigating propagative surface waves at the surface of a one-dimensional photonic crystal, known as Tamm surface waves, has increased due to their many applications in diverse fields, ranging from sensing and biosensing^8,9^ to integrated optics^10^. Excitation of TSW is due to TIR from external homogeneous layer on top of truncated photonic crystal and presence bandgap region of 1D photonic crystal.

Considering some advantages of TSWs over other surface waves such as SPPs, include lower propagation losses, versatility in material selection, enhanced sensing capabilities, the ability of TSWs to support both TE and TM polarizations, and mechanical, chemical, and thermal stability of TSW, these surface waves enable efficient light-matter interactions at the nanoscale and opening up ways for designing various integrated TSW nanophotonic devices^11–15^. Like any other wave, directionality of electric field vector of TSW forms its nature. Near field vector of Surface electromagnetic waves (SEWs) play a key role in applications of them in various fields, including data communication^16^, optical computing^17^, quantum information processing^18^, biosensors^19^, and biomedical imaging^20^. Many of these applications particularly rely on the transfer of energy or information between adjacent elements through near-field interactions. For example, in metasurfaces, engineering the nearfield coupling between neighboring individual elements in the nanostructures to achieve a wide range of functionalities, such as beam steering^21^, polarization conversion^22^, wavefront shaping^23^, and wavefront modulation^24^. Mentioned applications particularly clarify the importance of polarization state of nearfields of electromagnetic waves^25^. When electromagnetic waves are spatially confined at the nanoscale, the polarization state of localized evanescent waves or propagative SEWs get a different state from the polarization state of the exciting field. For instance, in total internal reflection of incoming transverse magnetic (TM) polarized light from the prism, in addition to transverse components of the incoming wave, a longitudinal field component parallel to the propagation direction emerges in SEW. This causes an elliptical polarization of the SEW with locked handiness to the propagation direction. The same condition takes place for TSW. Spin-momentum locking and intrinsic property of coupling between polarization states and phase gradients of TSW would shape non-trivial spatial structure for TSW. Topological features of SEWs including TSW, make growing interest for evanescent waves in field of structured light^26,27^. Without the possibility to map the polarization vectors of TSWs, effective usage of them in the structured light field looks ambiguous. As our understanding of SEWs is based on intuition from the free space waves, evaluation of polarization state of SEWs can finely tune their effectiveness in different applications. Evaluation of polarization of the light is a routine procedure in labs by known methods and apparatus like Stocks polarimeters. Direct measurement of the polarization state of TSW can be challenging due to the subwavelength localized nature of evanescent fields and decaying far from the interface. In fact, Conventional methods such as polarimetry methods, which rely on far-field, cannot be used for measuring the polarization vector of TSW as well as their electromagnetic field intensity distribution.

To solve this bottleneck and in order to study properties of evanescent fields, some methods are introduced based on nearfield interaction, such as scanning near field optical microscopy (SNOM)^28^, scattering by nano particles or nanostructures^29^, and spectroscopic measurements^30,31^. In the case of TSW, the energy would be attenuated by touch of absorbing material, depending on properties of both of evanescent field and absorbing material. To perform a vectorial characterization of TSW we consider an orientation sensitive interaction configuration of the polarization vector of TSW and absorbing atomic gas of rubidium.

In rubidium atomic gas, exerting an external magnetic field breaks the degeneracy of hyperfine energy states, this makes the relative orientation of polarization vector and direction of external magnetic field an important variable in resultant absorption spectrum. The main point in our proposed paradigm that makes quantitative characterization route simply applicable, is independency of absorption spectrum in several distinct geometrical configurations related to others. In other words, we can attribute type of abstract eigen spectrums (ES) to eigendirections (ED). Having in hand eigen spectrums we can linearly add up them to make spectrums in arbitrary geometrical configurations.

Alkali metals such as rubidium atomic gas have a wide range of applications in both fundamental and practical research such as slow and stored light^32^, generation of squeezed light sources^33^, atomic clocks^34^, metrology^35^, and quantum memories^36^. Also, the nonlinear optical effects in parity-time-symmetric atomic gases, showcase their potential as fundamental components for the development of innovative photonic devices capable of active light control at extremely low power levels^37^. Many efforts have been made to introduce evanescent fields-based light-matter interaction as a paradigm for designing chip-scale devices based on atomic gas cells for achieving strong light-matter interaction in new nanophotonic devices with enhanced performance in optical communications, quantum information processing, and other applications^38–40^.

Recently, many studies have investigated how atomic gases affect the behavior of electromagnetic waves within various photonic structures. For example, using the two-dimensional photonic crystals consisting of periodic dielectric rods immersed in a coherent atomic gas has been shown that mini-passbands, which could include nearly complete transmission peaks, can emerge within the stopbands of gas-free photonic crystals due to the strong frequency dispersion in the relative permittivity of the atomic gas^41^. Studies have also shown that the interaction between photonic structures based on photonic crystal waveguides with cold atoms with the intention of controlling and manipulating atomic interactions results in enhanced interactions between photons^42,43^.

In this paper, we employ the spectroscopic study in a hybrid TSW-atomic gas cell to characterize the polarization state of TSWs. The case of TSW spectroscopy of atomic gas is different from total internal reflection (TIR) spectroscopy. The results show TSW-Rb atoms coupling causes photons with properties exactly fulfilling selection rules of atomic transitions, survive absorption by TSW. This means an EIT-like phenomena in the case of TSW- Rb atoms coupling, despite simply absorption in the case of TIR-atom spectroscopy. Further, for the first time, by analyzing measurement results in Faraday and the Voigt configurations for TSW, we report the ratio between the longitudinal and the transverse components of the electromagnetic field of TSW. We believe by applying the magnetic field in three different directions relative to the direction of the transverse spin of the evanescent waves, this approach can be used for near-field vectorial imaging.

## Materials and methods

**Figure 1:**
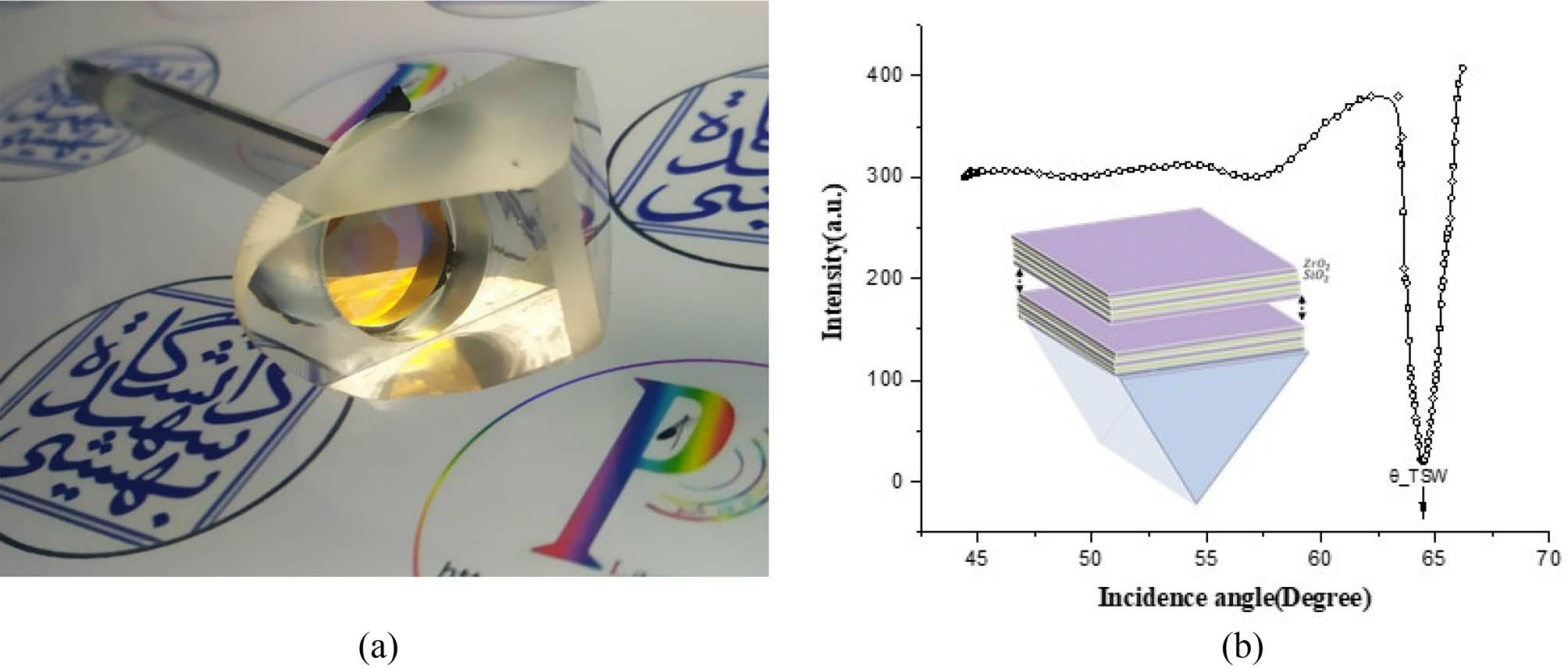
(**a**) Photograph of the hybrid 1D photonic crystal-atomic cell, (**b**) angular reflectivity of 795nm laser light from 1D photonic crystal in Kretschmann configuration. The TSW is excited when the incidence angle to the surface of the prism is 64.5°. Inset shows the schematic of 1D-PhC attached to the prism.

A photograph of the 1D photonic crystal-Rb atomic gas hybrid cell is illustrated in Fig. 1a. One-dimensional (1D) photonic crystal is fabricated of 12 periods of alternating high and low index layers and the extra last layer of SiO_2_, on the BK7 substrate by the e-beam deposition method (Bk7/(SiO_2_/ZrO_2_)^12^/SiO_2_). The central wavelength of the photonic crystal bandgap (PBG) is set to 808 nm so that the wavelength 795 nm corresponding to the 1D line of Rb is situated within the optical band gap of the structure. The structure of our photonic crystal is designed in such a way that only sustains TM-polarized TSW at this wavelength. The low-index and high-index material layers are SiO_2_ and ZrO_2_ with refractive indexes of 1.45 and 2.13, respectively, around the wavelength λ = 808 nm. The thicknesses of the ${\text{ZrO}}_{2}$ and ${\text{SiO}}_{2}$ layers are 94.83 nm and 139.31 nm.

A cylindrical glass chamber bonded to the substrate that photonic crystal was deposited on, such a way that PC is inserted inside the chamber and can have direct contact with contaminants of the chamber. In other words, we made 1D PC as an optical window of our hybrid cell. The hybrid cell was carefully cleaned and evacuated from the air and filled with a mixture of $3\times {10}^{-3}$ mbar Argon gas and little amount of natural isotope abundance of Rubidium metal and sealed through glass blowing technique to achieve a handmade TSW-atomic vapor cell. Truncated 1D-photonic crystal can sustain TSWs, which are electromagnetic waves confined at the interface between a periodic dielectric multilayer and atomic media. TSW excites in the Kretschmann configuration by total internal reflection of TM polarized 795nm laser light from a prism that is contacted to the substrate (BK7) of PC using an index-matching oil. In order to determine the angle of Tamm surface resonance (${\theta }_{BSW}$) the reflection spectrum of the sample was measured by sweeping the incident angle of light to the PC (Fig. 1b). To make the proper density of Rb gas, the cell is enclosed in a homemade oven and heated to a temperature of ∼ 70 °C. The 1D PC-atomic gas interface is illuminated by (TM) polarized 795 nm laser light to excite the TSW in wavelength equal to D1 transition line of Rb atoms. The measurement of reflected intensity from PC-atomic gas boundary performed by frequency modulation (FM) of laser light and lock-in amplifier detection. As a consequence, measured signals are the first derivative of real signals.

**Figure 2:**
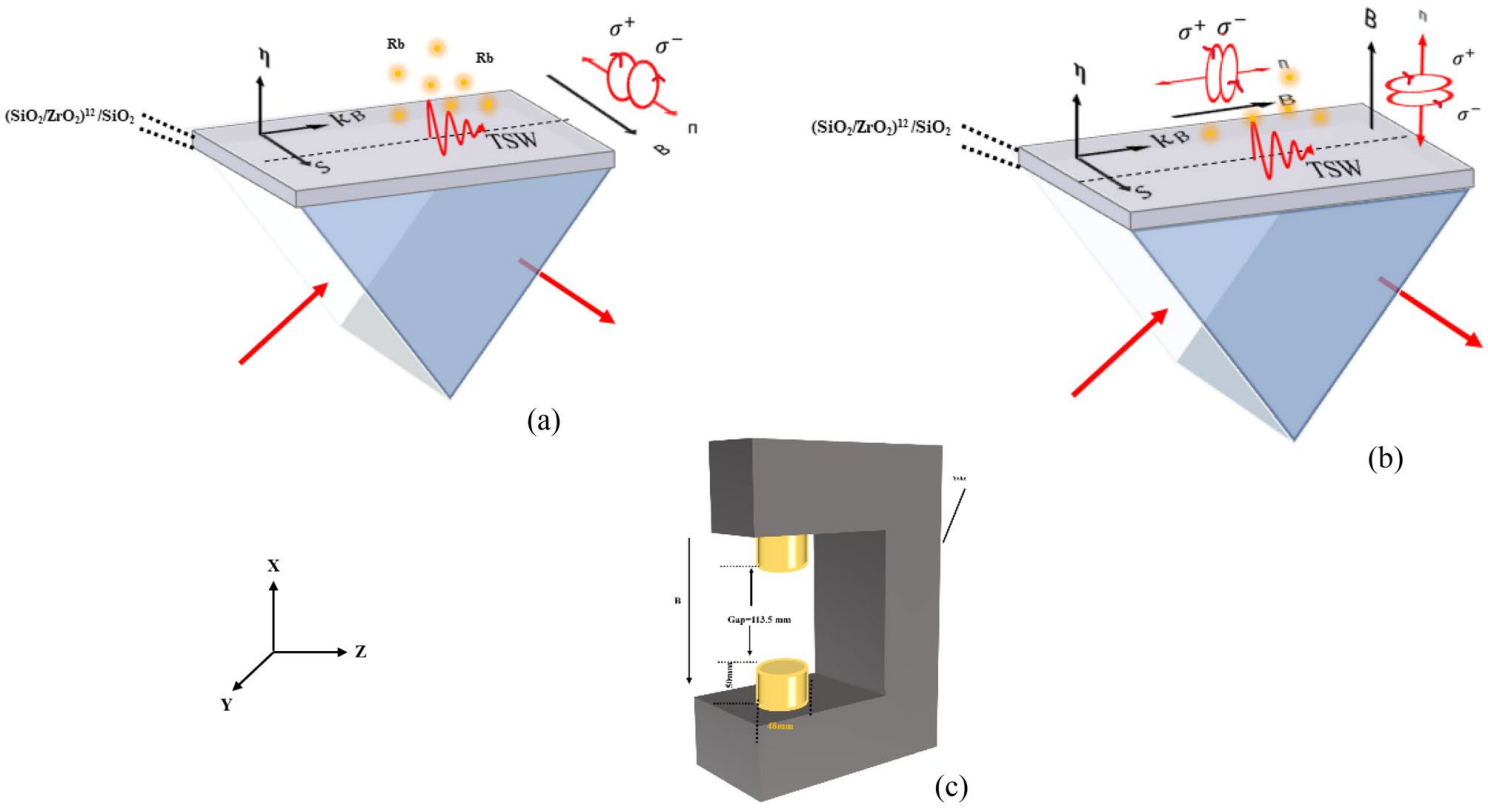
Faraday and Voigt geometry conditions for evanescent wave such Tamm surface waves: (**a**) Faraday condition for evanescent waves; schematics of the TSW-atomic system when the magnetic field is applied parallel to the s direction. (**b**) Voigt condition for evanescent waves; schematics of the TSW-atomic system when the magnetic field is applied transverse to the s direction. (**c**) Two disk neodymium magnets with Yoke; in both configurations, the hybrid TSW-atomic cell was set along a line equidistant between the two magnets where the magnetic field strength is 800 Gauss.

In the next step, 800 Gauss external static magnetic field was applied to the hybrid cell by inserting two disk neodymium magnets with Yok on the sides of the prism (Fig. 2c). In the absence of a magnetic field, Rb atomic energy levels are degenerate, meaning that different states with different total angular momentum (quantified by the quantum number J) have the same energy. However, when a magnetic field is introduced, makes the atomic gas anisotropic by introducing a preferred orientation for the atoms, therefore atomic energy levels split and the spectral lines modify which is as known Zeeman effect (Supplementary Information. S1, S4). Because our PC structure is made of ${\text{SiO}}_{2}$ and ${\text{ZrO}}_{2}$ and none of these materials have magnetic properties, the structure is not considered a magnetic photonic crystal.

We performed reflection spectrum measurements in the presence of an external magnetic field applied in distinct directions. The main idea is to measure the reflection spectrum of the TSW-atomic hybrid system in the Faraday and Voigt configurations, where the magnetic field vector is parallel or normal the direction of propagation light respectively.

## Results and discussion

As displayed schematically in Fig. 2a, b, we assume that the TSW propagates along the z-axis and the amplitude of its complex electric fields is written as:

1 $$ \vec{E} = \left( {\hat{x} - i\frac{\eta }{{k_{B} }}\hat{z}} \right)\exp \left( {ik_{B} z - \eta x} \right) $$

The π/2 phase difference of longitudinal $\left( {E_{z} = \frac{\eta }{{k_{B} }}} \right)$ and transverse (${E}_{x}=1$) components of the electric Tamm surface wave field implies effective elliptical polarization in the *xz* plane (Supplementary Information, S3). In this equation, ${k}_{B}$ is the longitudinal wave number (momentum), $\eta$ denotes the transverse wave number (exponential decay rate) and so complex wavevector of TSW is $\overrightarrow{k}={k}_{B}\widehat{z}+i \eta \widehat{x}$. For evanescent waves, the momentum of an evanescent wave (${\text{Re}}(\overrightarrow{k})$ is perpendicular to its decay direction ${\text{Im}}\left(\overrightarrow{k}\right)$. Also, to satisfy the transverse conditions ($\overrightarrow{k}.\overrightarrow{E}=0$), the two components of wave vector must have a phase difference of 90 degrees. Universal basic vectors for evanescent waves are expressed as a right-handed triplet consisting of ${\text{Re}}(\overrightarrow{k})$, ${\text{Im}}\left(\overrightarrow{k}\right)$ and $\overrightarrow{S}={\text{Re}}(\overrightarrow{k}) \times {\text{Im}}\left(\overrightarrow{k}\right)$ (transverse spin) which controls the direction of lateral forces. Spin-momentum locking in which the direction of momentum is locked into the direction of an intrinsic transverse spin, is an inherent feature of evanescent electromagnetic waves, including the Tamm surface wave^26^.

Considering this universal basis vectors, and the fact, that in the case of evanescent surface waves spin of the wave is perpendicular to the direction of motion of the surface wave, we can re-define the Faraday and Voigt geometry conditions for TSW as follows: the Faraday configuration, in which the direction of the transverse spin, S, of TSW and external magnetic field, B, is parallel (B perpendicular to the polarization ellipse), surface wave couples to transitions with ∆m_F_ =  ± 1 (σ^+^ and σ^−^, respectively).In Voigt configuration, in which the magnetic field is transverse to the direction of the spin, surface wave couples to ∆m_F_ = 0, ± 1 (transitions are coined π transitions and σ^+^ and σ^−^, respectively).

Note that the external magnetic field is not perpendicular to both components of the electric field of the surface wave simultaneously (Fig. 2b). More precisely, the polarization of the light influences the selection rules because the interaction between the light and magnetic field depends on the orientation of the atomic angular momentums relative to the direction of light polarization. As mentioned in the previous section, the Tamm surface wave has elliptical polarization in the xz plane, therefore, the component of the electric field of surface wave that is in the direction of the magnetic field drives π transitions. Also, the component of the electric field which is in the transverse of the magnetic field drives σ^+^ and σ^−^ transitions. Therefore, in the Faraday configuration for TSW, since the surface wave electric field has no component in the direction of the magnetic field, only ${\sigma }^{\pm }$ transitions are allowed. But in both proposed Voigt configurations $\left( {{\text{B}} \bot {\text{S}}\& {\text{B}}||,{\text{B}} \bot {\text{S}}\& {\text{ B}} \bot {\text{Re}} \left( {\vec{k}} \right)} \right)$, as shown in Fig. 2b, the component of the electric field of TSW that is in the direction of the magnetic field drives π transitions. Also, the component of the electric field which is in the transverse of the magnetic field drives σ^+^ and σ^−^ transitions.

Elliptical polarization of TSW (|E〉), can be represented as a superposition of right-handed circular polarization (|R〉) and left-handed circular polarization (|L〉) states. Mathematically, it can be expressed as:

2 $$ \left| E \right\rangle = \left| L \right\rangle \left\langle {L} \mathrel{\left | {\vphantom {L E}} \right. \kern-0pt} {E} \right\rangle + \left| R \right\rangle \left\langle {R} \mathrel{\left | {\vphantom {R E}} \right. \kern-0pt} {E} \right\rangle $$

So, if we measure the ratio of the σ^±^ transitions, we can specify polarization components of the electromagnetic field. Using Eq. (1) we can write Eq. (2) as,

3 $$ \left| E \right\rangle = {\raise0.7ex\hbox{$1$} \!\mathord{\left/ {\vphantom {1 {\sqrt 2 }}}\right.\kern-0pt} \!\lower0.7ex\hbox{${\sqrt 2 }$}}\left( {1 - \frac{\eta }{{k_{B} }}} \right)\left| L \right\rangle + {\raise0.7ex\hbox{$1$} \!\mathord{\left/ {\vphantom {1 {\sqrt 2 }}}\right.\kern-0pt} \!\lower0.7ex\hbox{${\sqrt 2 }$}}\left( {1 - \frac{\eta }{{k_{B} }}} \right)\left| R \right\rangle $$

According to this analysis, the ratio $$\frac{{1 + {\raise0.7ex\hbox{$\eta $} \!\mathord{\left/ {\vphantom {\eta {k_{B} }}}\right.\kern-0pt} \!\lower0.7ex\hbox{${k_{B} }$}}}}{{1 - {\raise0.7ex\hbox{$\eta $} \!\mathord{\left/ {\vphantom {\eta {k_{B} }}}\right.\kern-0pt} \!\lower0.7ex\hbox{${k_{B} }$}}}}$$ is given by the ratio between R and L basis.

The direction of the magnetic field determines the atomic quantization axis. In the case of free space transmission spectroscopy from a bulk rubidium vapor cell (we call it reference cell) at the Faraday configuration, the direction of the light-static magnetic field orientation limits hyperfine transitions between the ground and excited hyperfine atomic states to transitions with ${\Delta m}_{F}=\pm 1$, which is associated with ${\sigma }^{+}$ and ${\sigma }^{-}$ transitions. Further, in Voigt configuration transition with ${\Delta m}_{F}=0,\pm 1$ are allowed, which are associated with π and σ^±^ transitions. Therefore, by measuring the reference transmission spectrum of right/left-circularly polarized light (RCP/LCP) in Faraday configuration, we can determine the contributions of the ${\sigma }^{+}$ and ${\sigma }^{-}$ transitions of Rubidium, respectively. In fact, each of these transmission spectra could be supposed as an eigen spectrum. Therefore, we can express the unknown measured spectrum of the hybrid TSW-atomic gas in Faraday configuration based on the linear combination of these eigen spectrums.

**Figure 3:**
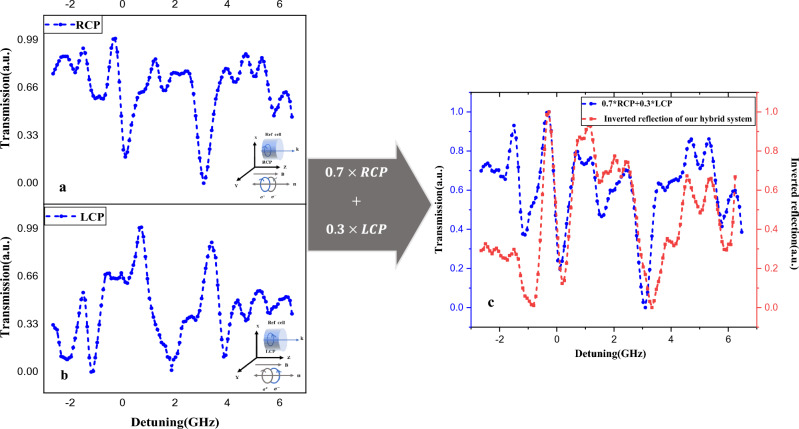
(**a**), (**b**) Transmission spectrums of reference atomic vapor cell for RCP and LCP light (eigen spectrums), respectively. The magnetic field is applied in free space Faraday configuration. The schematic of these measurements is shown inset of these spectrums, (**c**) Spectrum of hybrid TSW-atomic structure (red curve) when magnetic field is applied parallel to the s direction (Faraday configuration for evanescent waves) and normalized spectra of summed right- and left-handed circular transitions with ratios of 7:3 (blue curve).

By adjusting the incident light angle to the prism at the angle of Tamm surface resonance (${\theta }_{BSW}={64.5}^{^\circ }$), we measured the reflection spectra of the TSW-atomic hybrid system in the presence of the external magnetic field. In the first step, according to Fig. 2b, we apply a magnetic field to the hybrid system parallel to the direction of the spin (Faraday configuration for evanescent waves). The measured reflection spectrum from the interface of the photonic crystal and the atomic vapor is represented by the red spectrum in Fig. 3c.

As mentioned above, in the Faraday configuration for evanescent waves ($B||S$) we expect only transitions with ${\Delta m}_{F}=\pm 1$ are allowed. To prove this assumption and to determine the contribution of right and left-handed circular polarization to the measured spectrum, as depicted in Fig. 3a, b, we measured the transmission spectra of the reference atomic vapor cell in the cases of right and left-handed circularly polarized light propagating in atomic media where the applied magnetic field was parallel to the wave vector of light (free space Faraday configuration). As stated in the previous section, according to selection rules, the left and right circular polarized light drives σ^−^ and σ^+^ transitions, respectively.

As we expected from the two coupled springs model^44^, the coupling of surface wave (TSW) and hyperfine transitions of the Rb atom causes photon energy to survive the absorption by surface resonance, when two resonances have the same resonance frequency^45^. Resonant coupling renders TSW-atom hybrid structure medium transparent within a narrow spectral range around absorption lines of Rubidium, so we call this phenomenon EIT-like resonance of TSW-atom.

This induced transparency is proof of the existence of surface waves in the interface of 1D PC in the presence of atomic gas. As the difference between the absorption of the hybrid cell and reference cell is that in detuning equal to Rb transitions we observe Induced transparency in spite of photon attenuation thus, for a better comparison of these two spectra, the measurement spectrum of the hybrid system has been inverted (measure intensity multiplied by − 1). As evidenced in Fig. 3, we summed the normalized spectra of right- and left-handed circular transitions with ratios of 7:3. Clearly, we find a striking similarity between the spectral lines derived from 0.7*RCP + 0.3*LCP combination (depicted as the blue line in Fig. 3c) and the spectral lines measured in the hybrid system (illustrated as the red line in Fig. 3c). The dissimilar magnitudes between the two graphs in Fig. 3c is caused by the logical difference between the measurement and the ideal percentage of LCP and RCP light which must be used. Therefore, the ratio between left-handed and right-handed circular polarization is obtained as 7:3, which is equal to $$\frac{{1 + {\raise0.7ex\hbox{$\eta $} \!\mathord{\left/ {\vphantom {\eta {k_{B} }}}\right.\kern-0pt} \!\lower0.7ex\hbox{${k_{B} }$}}}}{{1 - {\raise0.7ex\hbox{$\eta $} \!\mathord{\left/ {\vphantom {\eta {k_{B} }}}\right.\kern-0pt} \!\lower0.7ex\hbox{${k_{B} }$}}}}$$, according to Eq. (2). Now, we obtained the ratio between the TSWs electric field components and TSW wavenumbers in the x and z directions as follows:

$$ \frac{{E_{z} }}{{E_{x} }} = \frac{{k_{x} }}{{k_{z} }} = {\raise0.7ex\hbox{$\eta $} \!\mathord{\left/ {\vphantom {\eta {k_{B} }}}\right.\kern-0pt} \!\lower0.7ex\hbox{${k_{B} }$}} = \frac{2}{5} $$

This means the Tamm surface wave electric field with elliptical polarization rotates in the xz plane while the ratio of its longitudinal and transverse electric field components is equal to 2/5. For other surface waves, such as surface plasmon polaritons, considering that the depth of penetration and propagation length of them is less than Tamm surface waves, the ratio ${k}_{B}$/η which is proportional to depth of penetration expected to be lower.

**Figure 4:**
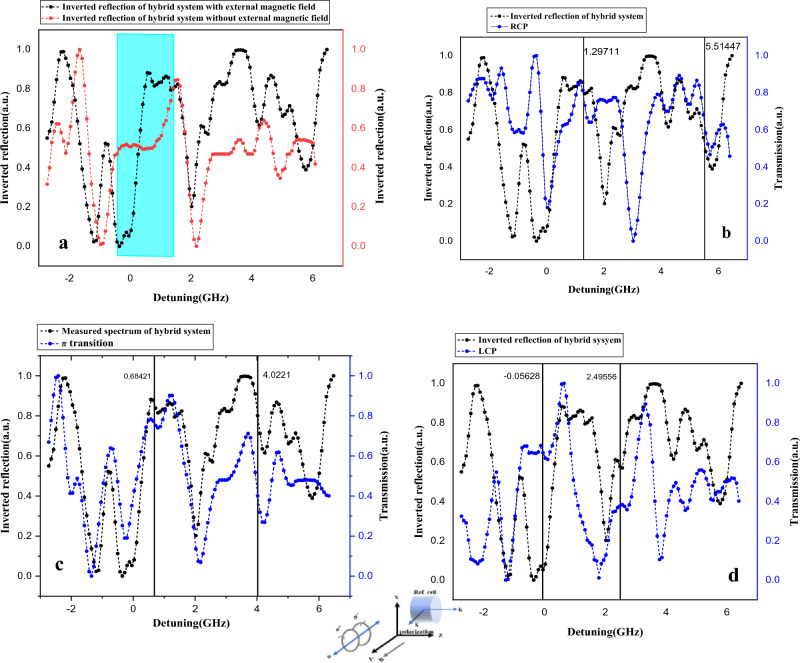
(**a**) Measured spectrum of the hybrid TSW-atomic system in Voigt configuration for evanescent wave with external magnetic field (black dash curve) and without external magnetic field (red dash curve). The magnetic field is applied perpendicular to the spin direction, (**b**), (**c**), (**d**) Comparison of the transmission spectra including ${\sigma }^{+}$, $\pi$ and ${\sigma }^{-}$ transitions(eigenspectrum) respectively with the spectrum measured from the hybrid TSW-atomic system. The vertical lines in each figure indicate the corresponding transitions in the two spectra.

In the second step, we measure the reflection spectrum of a hybrid cell with and without the presence of a magnetic field in Fig. 4a, the black curve represents the reflection spectrum of our hybrid system in the Voigt configuration for evanescent fields according to the schematic of Fig. 2b and the red curve represents the reflection spectrum of our hybrid system without the presence of a magnetic field. Comparing these two curves shows the impact of the magnetic field on the transition marked by the blue range which has split into three transitions due to Zeeman splitting.

In Voigt configuration, the magnetic field can be applied in the direction of TSW propagation or the direction of TSW decay. As mentioned in the previous section, in this geometry we expect that three transitions ($\Delta {m}_{F}=0,\pm 1$) are allowed because the electromagnetic field component of the Tamm surface wave which is perpendicular to the atomic quantization axis (magnetic field axis) drives σ^+^ and σ^−^ transitions, and the field component that is aligned with the atomic quantization axis, drives the π transitions.

As shown in Fig. 4, we were able to behold such an effect. To express this observation more precisely and prove this claim, we first measured free space transmission spectrum of the reference atomic vapor cell by incoming TE Linear polarized laser light in Voigt configuration. As the schematic of Fig. 4c shows, the incident TE polarized light only excites the $\pi$ atomic transitions. Therefore, the magnetically affected spectrum for reference atomic vapor cell only includes $\pi$ transitions (black dash curve). Also, black dash curves Fig. 4b, d represent measured transmission spectra which include ${\sigma }^{+}$ and ${\sigma }^{-}$ transitions respectively. The method of measuring the spectrum of ${\sigma }^{\pm }$ transitions was mentioned in the previous section. Next, we compared the magneto-optical response of the hybrid system with each of these spectra, which include ${\sigma }^{+}$, $\pi$ and ${\sigma }^{-}$ transitions respectively. In Fig. 4a, the blue highlighted part of the spectrum shows all three transitions in this hybrid system are allowed. We have taken a systematic approach to compare the magneto-optical response of our hybrid system with the spectra from the reference atomic vapor cell under different polarization and the same magnetic field strength.

The presence of ${\sigma }^{+}$, $\pi$ and ${\sigma }^{-}$ transitions and their alignment with the blue-highlighted part of the hybrid system's spectrum demonstrates the effects of the magnetic field on the spectral transitions, providing strong experimental evidence to support our claims.

## Conclusion

In summary, we have proposed a scheme to investigate the polarization of Tamm surface waves using the coupling of TSW and hot atoms. For this purpose, we have studied the coupling of TSW and the hot vapor of Rb in the presence of an external magnetic field. As the first result of these studies, considering the intrinsic property of evanescent waves, spin-momentum locking, we redefined the Faraday and Voigt configuration based on the orientation of the external magnetic field and the inherent transverse spin of evanescent waves, especially TSW. By evanescent spectroscopic of the TSW-atomic hybrid system in the Faraday configuration of the evanescent wave, we have shown the ellipticity of Tamm surface wave polarization and measured the ratio between the TSW electric field transverse and longitude components. As a result, this method is appealing toward mapping the electromagnetic polarization components and near-field vectorial imaging which is an important tool that enables the visualization and analysis of the vector nature of electromagnetic fields at nanometer scales, opening up a wide range of scientific and technological possibilities.

### Supplementary Information

Supplementary Information.

## Data Availability

Data other than presented here may be obtained from the authors upon request from the corresponding author.
