# Supplementary material for: Vectorial characterization of surface wave via one-dimensional photonic-atomic structure
Source: Sci Rep. 2023 Dec 8;13:21783. doi: 10.1038/s41598-023-49324-6 (PMC10709312; doi:10.1038/s41598-023-49324-6)
Supplement: Supplementary file 1 — Supplementary Information. [file 41598_2023_49324_MOESM1_ESM.pdf]

Supplementary material:

Vectorial characterization of surface wave via one-dimensional photonic-atomic structure

M. Asadolah Salmanpour, M. Mosleh, S. M. Hamidi*

Magneto-plasmonic Lab, Laser and Plasma Research Institute, Shahid Beheshti University, Tehran, Iran.
*Corresponding author: m_hamidi@sbu.ac.ir

A. permittivity tensor for Rb gas in the presence of a magnetic field:

The permittivity tensor for Rb gas in the presence of a magnetic field can be obtained through theoretical modeling or experimental measurements. It allows for a more comprehensive description of the optical properties of Rb gas in the presence of a magnetic field, taking into account the gyrotropic effects. It enables the calculation of quantities like the refractive index, which can vary with the direction of propagation and the polarization state of the incident light. It depends on the specific parameters of the gas, such as the magnetic field strength, temperature, pressure, and atomic properties. In Rb hot vapor as magneto-optic material, the presence of an applied magnetic field along the z-axis, modifies dielectric tensor ε of hot vapor as gyrotropic form [1]:

$$\varepsilon = \varepsilon_0 I + \sum_{\pm,0} \mathcal{X}_i |a_i\rangle\langle a_i| = \varepsilon_0 I + \chi_+ |\sigma_+ >< \sigma_+| + \chi_- |\sigma_- >< \sigma_-| + \chi_0 |\pi >< \pi| \quad \text{(eq s.1)}$$

Where $|\sigma_- >= {}^{1}/_{\sqrt{2}} \begin{pmatrix} 1 \\ -i \\ 0 \end{pmatrix}$, $|\sigma_+ >= {}^{1}/_{\sqrt{2}} \begin{pmatrix} 1 \\ +i \\ 0 \end{pmatrix}$, $|\pi >= \begin{pmatrix} 0 \\ 0 \\ 1 \end{pmatrix}$

$$\varepsilon = \varepsilon_0 \begin{bmatrix} 1 & 0 & 0 \\ \vdots & 1 & 0 \\ 0 & 0 & 1 \end{bmatrix} + \chi_+ \begin{bmatrix} 1 & -i & 0 \\ i & 1 & 0 \\ 0 & 0 & 0 \end{bmatrix} + \chi_- \begin{bmatrix} 1 & +i & 0 \\ -i & 1 & 0 \\ 0 & 0 & 0 \end{bmatrix} + \chi_0 \begin{bmatrix} 0 & 0 & 0 \\ 0 & 0 & 0 \\ 0 & 0 & 1 \end{bmatrix}$$

$$= \begin{bmatrix} \frac{\varepsilon_0}{2}(2 + \chi_+ + \chi_-) & \frac{\varepsilon_0}{2} i(\chi_- - \chi_+) & 0 \\ -\frac{\varepsilon_0}{2} i(\chi_- - \chi_+) & \frac{\varepsilon_0}{2}(2 + \chi_+ + \chi_-) & 0 \\ 0 & 0 & \varepsilon_0(1 + \chi_0) \end{bmatrix} \quad \text{(eq s.2)}$$

where the polarizable Polarizability tensor elements $\chi\pm$ and $\chi 0$ are related to susceptibility of atomic transitions with angular momentum ± 1 and 0(are associated with $\sigma^{\pm}$and π transitions.)

The wave equation for an electromagnetic wave propagating in a non-magnetic dielectric medium based on Maxwell's equations can be derived. Here's the resulting wave equation:

$$\mathbf{k} \times (\mathbf{k} \times \mathbf{E}) + \frac{1}{\varepsilon_0}\left(\frac{\omega}{c}\right)^2 \varepsilon.\mathbf{E} = 0 \quad \text{(eq s.3)}$$

assuming that the wavevector of propagative light makes an angle with the z-axis, direction of the external magnetic field, and with defining complex refractive index n as $n^2 = (\frac{c}{\omega})^2\mathbf{k}.\mathbf{k}$, we can write matrix form of wave equation as:

$$\begin{bmatrix} \frac{\varepsilon_0}{2}(2+\chi_+ +\chi_-) - n^2\cos\theta^2 & \frac{\varepsilon_0}{2} i(\chi_- - \chi_+) & n^2\cos\theta\sin\theta \\ -\frac{\varepsilon_0}{2} i(\chi_- - \chi_+) & \frac{\varepsilon_0}{2}(2+\chi_+ +\chi_-) - n^2 & 0 \\ n^2\cos\theta\sin\theta & 0 & \varepsilon_0(1+\chi_0) - n^2\sin\theta^2 \end{bmatrix} \begin{bmatrix} E_x \\ E_y \\ E_z \end{bmatrix} = 0 \quad \text{(eq s.4)}$$

for Faraday configuration, where $\mathbf{k}||\mathbf{B}$, $(\boldsymbol{\theta} = \mathbf{0})$, by solving the eigenvalue problem for the dispersion matrix we obtained the refractive index and eigenvectors as

$$n_1 = \sqrt{\varepsilon_0(1+\chi_+)} \qquad \text{(eq s.5)}$$

$$\text{and} \quad n_2 = \sqrt{\varepsilon_0(1+\chi_-)} \quad \text{(eq s.6)}$$

$$e_1 = \frac{1}{\sqrt{2}}\begin{pmatrix} 1 \\ -i \\ 0 \end{pmatrix} \quad \text{(eq s.7)}$$

$$\text{and} \quad e_2 = \frac{1}{\sqrt{2}}\begin{pmatrix} 1 \\ i \\ 0 \end{pmatrix} \text{(eq s.8)}$$

It is clear that $\sigma^{\pm}$ transitions are excited by right/left circularly polarised light respectively. In the Faraday configuration, no polarized light cannot excite π transitions (Figure S1 a).

In Voigt geometry, where the magnetic field axis is transverse to the light propagation axis $(\mathbf{k} \perp \mathbf{B}, (\boldsymbol{\theta} = \frac{\boldsymbol{\pi}}{\mathbf{2}}))$, refractive index and eigenvectors obtain as:

$$n_1 = \sqrt{\varepsilon_0 \frac{2(1+\chi_+ +\chi_- +\chi_+\chi_-)}{(2+\chi_+ +\chi_-)}} \quad \text{(eq s.9)}$$

$$\text{and} \quad n_2 = \sqrt{\varepsilon_0(1+\chi_0)} \quad \text{(eq s.10)}$$

$$e_1 = \frac{1}{\sqrt{\frac{2\chi_+}{\chi_+ - \chi_-}}}\begin{pmatrix} 1 \\ i\frac{(2+\chi_+ +\chi_-)}{(\chi_- - \chi_+)} \\ 0 \end{pmatrix} \text{(eq s.11)}$$

$$\text{and} \quad e_2 = \begin{pmatrix} 0 \\ 0 \\ 1 \end{pmatrix} \text{(eq s.12)}$$

The refractive indices (n_1 and n_2) and their associated eigenvectors correspond to different types of atomic transitions. n_1 corresponds to both σ+ and σ- transitions and n_2 corresponds to only π transitions. e_1 represents elliptically polarized light in the plane perpendicular to $\vec{B}$, and it equally drives both σ+ and σ- transitions. More clearly Let's assume that the incident light is linearly polarized along the y-axis in the laboratory frame. In the laboratory frame, this linear polarization can be described as a superposition of two circularly polarized components with equal amplitudes and opposite handedness, rotating in the X-Y plan. Therefore, the linearly polarized light along the y-direction in the laboratory frame can be broken down into equal circular components in the atomic frame, which drive both σ+ and σ- transitions in Voigt geometry due to the orientation of the atomic quantization axis with respect to the external magnetic field. e2 is associated with the field component that is parallel to the magnetic field (E_z|| B) and drives π transitions (Figure S1 b, c).

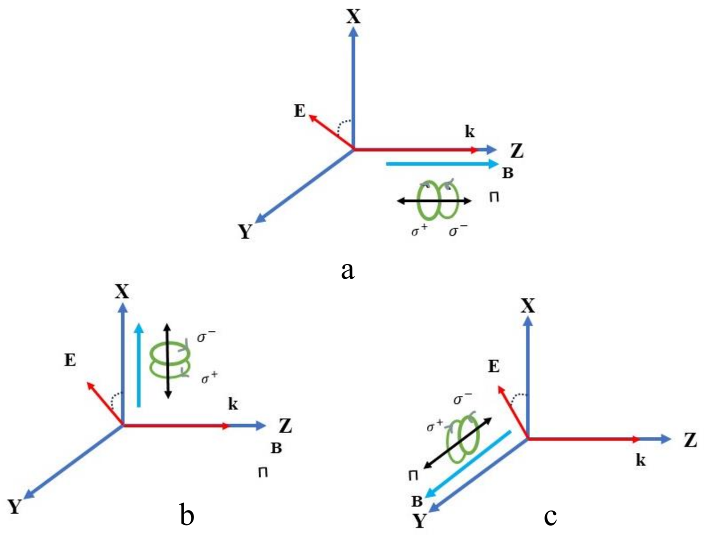

Figure S1: (a) Faraday and (b, c) Voight configuration for free space

As a general conclusion, if the electromagnetic field of incident light has a component perpendicular to the atomic quantization axis (magnetic field axis), it drives σ+ and σ- transitions. The field component that is aligned with the atomic quantization axis, drives the π transitions. As the Tamm surface wave has elliptical polarization in the xz plane, therefore, the component of the electric field of the incident light that is in the direction of the magnetic field drives π transitions. Also, the component of the electric field which is in the transverse of the magnetic field drives σ+ and σ- transitions (Figure S2).

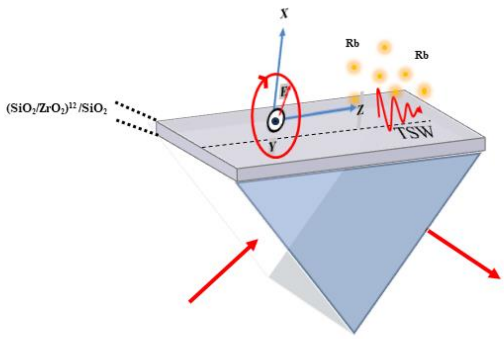

Figure S2: Elliptical polarization of Tamm surface wave

B. Theoretical foundation for Ellipticity of polarization of the Tamm surface wave (TSW)

This analysis will provide a solid theoretical underpinning for the polarization state of the Tamm surface wave in our system [2].

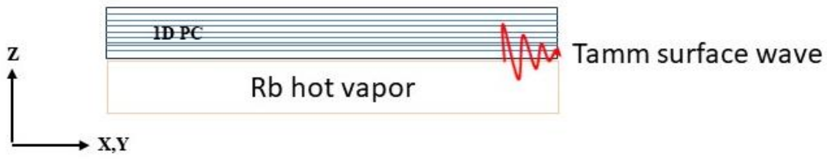

Figure S3: schematic of our system

Considering our system; the periodically nonhomogeneous photonic crystal which is in the direction normal to the interface, type A, occupies the half-space z > 0, and the anisotropic Rb vapor, type B, occupies the half-space z < 0, as shown in Figure S3. The constitutive relations are stated as

$$\mathbf{D}(\mathbf{r}) = \begin{cases} \varepsilon^{A}(z).\mathrm{E}(r) & z > 0 \\ \varepsilon^{B}.\mathrm{E}(r) & z < 0 \end{cases} \quad \text{(eq s.13)}$$

$$\mathbf{B}(\mathbf{r}) = \begin{cases} \mu_0^{A}.\mathrm{H}(r) & z > 0 \\ \mu_0^{B}.\mathrm{H}(r) & z < 0 \end{cases} \quad \text{(eq s.14)}$$

field phasors of p-polarized Tamm surface wave where $e_x(z), h_y(z), e_z(z) \neq 0$:

$$\mathbf{E}(\mathbf{r}) = [e_x(z)\hat{x} + e_z(z)\hat{z}]\exp(iqx) \quad \text{(eq s.15)}$$

$$\mathbf{H}(\mathbf{r}) = h_y(z)\hat{y}\exp(iqx) \quad \text{(eq s.16)}$$

Where q is complex wavenumber of TSW. By using Maxwell equations:

$$\frac{de_x(z)}{dz} = i\omega\left[\frac{q}{\omega}e_z(z) + \mu_0 h_y(z)\right] \qquad \text{(eq s. 17)}$$

$$\frac{dh_y(z)}{dz} = i\omega[\varepsilon_{xx}e_x(z) + \varepsilon_{xz}e_z(z)] \qquad \text{(eq s. 18)}$$

$$e_z(z) = -\frac{\varepsilon_{zx}}{\varepsilon_{zz}}e_x(z) - \frac{q}{\omega\varepsilon_{zz}}h_y(z) \qquad \text{(eq s. 19)}$$

$$e_z(z) = \begin{cases} \dfrac{-q}{\omega\varepsilon^A(z)}h_y(z) & z > 0 \qquad \text{(eq s. 20)} \\ -\dfrac{\varepsilon^B_{zx}}{\varepsilon^B_{zz}}e_x(z) - \dfrac{q}{\omega\varepsilon^B_{zz}}h_y(z) & z < 0 \qquad \text{(eq s. 21)} \end{cases}$$

For $Z > 0$, we insert eq s. 20 in eq s. 17 and eq s. 18 then obtain:

$$\frac{de_x(z)}{dz} = i\omega\left[\frac{q}{\omega}\frac{-q}{\omega\varepsilon^A(z)}h_y(z) + \mu_0 h_y(z)\right] \qquad \text{(eq s. 22)}$$

$$\frac{dh_y(z)}{dz} = i\omega[\varepsilon^A(z)e_x(z)] \qquad \text{(eq s. 23)}$$

For $Z < 0$, we insert eq s. 21 in eq s. 17 and eq s. 18 then obtain:

$$\frac{de_x(z)}{dz} = -iq\frac{\varepsilon^B_{zx}}{\varepsilon^B_{zz}}e_x(z) + \left(-i\frac{q^2}{\omega\varepsilon^B_{zz}} + i\omega\mu_0\right)h_y(z) \qquad \text{(eq s. 24)}$$

$$\frac{dh_y(z)}{dz} = \left(i\omega\varepsilon_{xx} - i\omega\frac{\varepsilon^B_{xz}\,\varepsilon^B_{zx}}{\varepsilon^B_{zz}}\right)e_x(z) - i\frac{q\,\varepsilon^B_{xz}}{\varepsilon^B_{zz}}h_y(z) \qquad \text{(eq s. 25)}$$

The pair of eqs s. 22, 23 (as well as eqs s. 20, 21) yields the 2 × 2 matrix ordinary differential:

$$\frac{d}{dz}\left[\underline{f_p}(z)\right] = i\left[\underline{P}\right].\left[\underline{f_p}(z)\right] \qquad \text{(eq s. 26)}$$

where the column 2-vector

$$\left[\underline{f_p}(z)\right] = \begin{bmatrix} e_x(z) \\ h_y(z) \end{bmatrix} \qquad \text{(eq s. 27)}$$

and the matrix

$$P = \begin{bmatrix} -q\frac{\varepsilon_{zx}}{\varepsilon_{zz}} & (-\frac{q^2}{\omega\varepsilon_{zz}} + \omega\mu_0) \\ \left(\omega\varepsilon_{xx} - \omega\frac{\varepsilon_{xz}\ \varepsilon_{zx}}{\varepsilon_{zz}}\right) & -\frac{q\ \varepsilon_{xz}}{\varepsilon_{zz}} \end{bmatrix} \qquad \text{(eq s. 28)}$$

For Z > o

$$P^A(z) = \begin{bmatrix} 0 & \left(-\frac{q^2}{\omega\varepsilon^A(z)} + \omega\mu_0\right) \\ \omega\varepsilon^A(z) & 0 \end{bmatrix} \qquad \text{(eq s. 29)}$$

For Z < o and with considering dielectric tensor ε of hot vapor:

$$P = \begin{bmatrix} -q\frac{\varepsilon_{zx}^B}{\varepsilon_{zz}^B} & (-\frac{q^2}{\omega\varepsilon_{zz}^B} + \omega\mu_0) \\ \left(\omega\varepsilon_{xx}^B - \omega\frac{\varepsilon_{xz}^B\ \varepsilon_{zx}^B}{\varepsilon_{zz}^B}\right) & -\frac{q\ \varepsilon_{xz}^B}{\varepsilon_{zz}^B} \end{bmatrix} \qquad \text{(eq s. 30)}$$

,

$$\varepsilon_{Rb} = \begin{bmatrix} \frac{\varepsilon_0}{2}(2+\chi_+ +\chi_-) & \frac{\varepsilon_0}{2}i(\chi_- - \chi_+) & 0 \\ -\frac{\varepsilon_0}{2}i(\chi_- - \chi_+) & \frac{\varepsilon_0}{2}(2+\chi_+ +\chi_-) & 0 \\ 0 & 0 & \varepsilon_0(1+\chi_0) \end{bmatrix} \qquad \text{(eq s. 31)}$$

$$P^B(\chi_+, \chi_-, \chi_0) = \begin{bmatrix} 0 & \left(-\frac{q^2}{\omega\varepsilon_0(1+\chi_0)} + \omega\mu_0\right) \\ \omega\frac{\varepsilon_0}{2}(2+\chi_+ +\chi_-) & 0 \end{bmatrix} \qquad \text{(eq s. 32)}$$

obeys the 2 × 2 matrix ordinary differential equation:

$$\frac{d}{dz}\left[\underline{f_p}(z)\right] = \begin{cases} i[P^A(z)].\left[\underline{f_p}(z)\right] & z > 0 \\ i[P^B(\chi_+, \chi_-, \chi_0)].\left[\underline{f_p}(z)\right] & z < 0 \end{cases} \qquad \text{(eq s. 33)}$$

We solve these equations (eq s. 33) near the interface of two media to demonstrate the elliptical polarization of Tamm surface waves.

For Z < o and using eq s. 21:

$$e_x(z) = A_0 \exp\left(\sqrt{q^2 - \omega^2\mu_0\varepsilon_0(1+\chi_0)}\sqrt{\frac{2+\chi_+ + \chi_-}{2(1+\chi_0)}}\, z\right) \qquad \text{(eq s. 34)}$$

$$h_y(z) = iA_0\, \omega\varepsilon_0 \frac{\sqrt{2(2+\chi_+ + \chi_-)(1+\chi_0)}}{2\sqrt{q^2 - \omega^2\mu_0\varepsilon_0(1+\chi_0)}} \exp\left(\sqrt{q^2 - \omega^2\mu_0\varepsilon_0(1+\chi_0)}\sqrt{\frac{2+\chi_+ + \chi_-}{2(1+\chi_0)}}\, z\right) \qquad \text{(eq s. 35)}$$

$$e_z(z) = \frac{iA_0 q}{\sqrt{q^2 - \omega^2\mu_0\varepsilon_0(1+\chi_0)}}\sqrt{\frac{2+\chi_+ + \chi_-}{2(1+\chi_0)}} \exp\left(\sqrt{q^2 - \omega^2\mu_0\varepsilon_0(1+\chi_0)}\sqrt{\frac{2+\chi_+ + \chi_-}{2(1+\chi_0)}}\, z\right) \qquad \text{(eq s. 36)}$$

For z>0 and using eq s. 20

$$e_x(z) = B_0 \exp\left(-i\sqrt{q^2 - \omega^2\mu_0\varepsilon^A(z)}z\right) \qquad \text{(eq s. 37)}$$

$$h_y(z) = iB_0 \frac{\omega\varepsilon^A(z)}{\sqrt{q^2 - \omega^2\mu_0\varepsilon^A(z)}} \exp\left(-i\sqrt{q^2 - \omega^2\mu_0\varepsilon^A(z)}z\right) \qquad \text{(eq s. 38)}$$

$$e_z(z) = \frac{iB_0 q}{\sqrt{q^2 - \omega^2\mu_0\varepsilon^A(z)}} \exp\left(-i\sqrt{q^2 - \omega^2\mu_0\varepsilon^A(z)}z\right) \qquad \text{(eq s. 39)}$$

eqs s. 34, 36 show that in the TM polarization case, the electric field of TEW has two components that have a phase shift equal to π/2 with respect to each other. This results in elliptical polarization in the plane of incidence.

C. Effect of magnitude of external magnetic fields on spectral lines shift

In weak magnetic fields, the Zeeman splitting is relatively small, and the spectral lines associated with different sublevels may be overlapped. In strong magnetic fields, the Paschen-Back effect becomes significant. The energy levels of the atom can undergo substantial splitting, leading to distinct spectral lines. Frequency shift of these lines depends on magnitude of external magnetic fields [3]. For example, the level hyperfine structure of the D1 line of 87Rb in the presence of a magnetic field is shown in Fig. 4S in the weak field (anomalous Zeeman) regime through the hyperfine Paschen-Back regime. In the Paschen-Back regime in both ground and excited states, one of the splitting of levels in F=2 has a redshift frequency compared to three other

levels. This level contributes in transition of F=1, m_F=-1(ground state;$5^2S_{1/2}$) to F=2, m_F=-2 (excited state; $5^2P_{1/2}$). Δm_F=-1 so this transition is associated with σ- transitions. This transition results in a distinct spectral line in the lower detuning frequency of the measured spectrum of the hybrid system. According to Fig. 2b (in manuscript), in the first Voight configuration where B is parallel to K_B, the transverse electric field component of light drives σ- transitions. In the second Voight configuration where B is parallel to **η**, the longitude electric field component of light drives σ- transitions. Therefore, each of the measured spectral lines in configurations provides us with unique information about the electric field components of the surface wave.

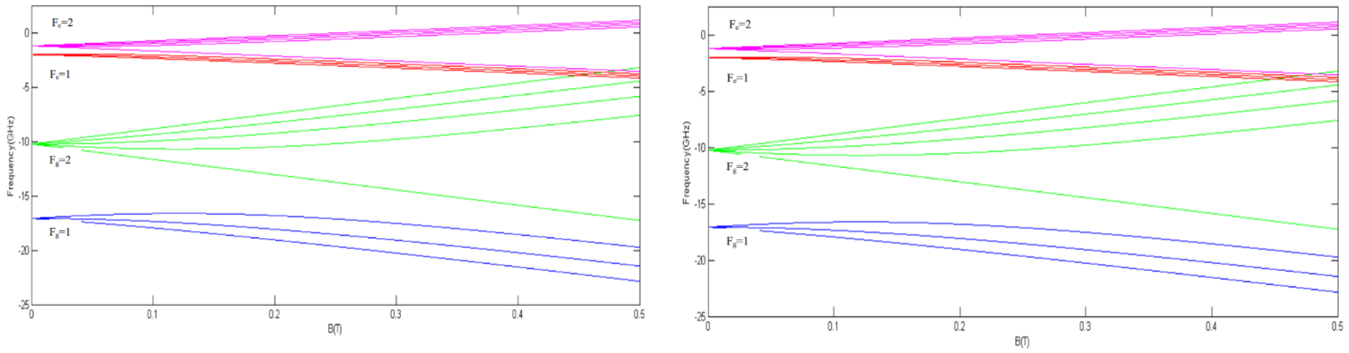

Figure S4: (a) 87Rb 52S1/2 (ground) level hyperfine structure in an external magnetic field. (b) 87Rb 52P1/2 (D1 excited) level hyperfine structure in an external magnetic field.

References:

[1] Rotondaro MD, Zhdanov BV, Knize RJ. Generalized treatment of magneto-optical transmission filters. JOSA B. 32(12):2507-13, (2015).

[2] Polo J, Mackay T, Lakhtakia A. Electromagnetic surface waves: a modern perspective. Newnes, (2013).

[3] Steck DA. Rubidium 87 D line data.
